# NKX2–1 expression as a prognostic marker in early-stage non-small-cell lung cancer

**DOI:** 10.1186/s12890-017-0542-z

**Published:** 2017-12-13

**Authors:** Jorge Moisés, Alfons Navarro, Sandra Santasusagna, Nuria Viñolas, Laureano Molins, José Ramirez, Jeisson Osorio, Adela Saco, Joan Josep Castellano, Carmen Muñoz, Sara Morales, Mariano Monzó, Ramón María Marrades

**Affiliations:** 1 Department of Pneumology, Institut Clínic Respiratori (ICR), Hospital Clínic de Barcelona, University of Barcelona, IDIBAPS, CIBER de Enfermedades Respiratorias (CIBERES), Barcelona, Spain; 2 Molecular Oncology and Embryology Laboratory, Human Anatomy Unit, School of Medicine, University of Barcelona, IDIBAPS, Casanova 143, 08036 Barcelona, Spain; 3 Department of Medical Oncology, Institut Clínic de Malalties Hematològicas i Oncològiques (ICMHO), Hospital Clínic de Barcelona, University of Barcelona, IDIBAPS, Barcelona, Spain; 4 Department of Thoracic Surgery, Institut Clínic Respiratori (ICT), Hospital Clínic de Barcelona, University of Barcelona, Barcelona, Spain; 5 Department of Pathology, Centre de Diagnòstic Biomèdic (CDB), Hospital Clínic de Barcelona, University of Barcelona, IDIBAPS, CIBERES, Barcelona, Spain

**Keywords:** NKX2–1, microRNA, TP53, NSCLC, miR-365, miR-33a

## Abstract

**Background:**

NKX2–1, a key molecule in lung development, is highly expressed in non-small cell lung cancer (NSCLC), particularly in lung adenocarcinoma (ADK), where it is a diagnostic marker. Studies of the prognostic role of NKX2–1 in NSCLC have reported contradictory findings. Two microRNAs (miRNAs) have been associated with NKX2–1: miR-365, which targets NKX2–1; and miR-33a, which is downstream of NKX2–1. We have examined the effect of NKX2–1, miR-365 and miR-33a on survival in a cohort of early-stage NSCLC patients and in sub-groups of patients classified according to the mutational status of TP53, KRAS, and EGFR.

**Methods:**

mRNA and miRNA expression was determined using TaqMan assays in 110 early-stage NSCLC patients. TP53, KRAS, and EGFR mutations were assessed by Sanger sequencing.

**Results:**

NKX2–1 expression was upregulated in never-smokers (*P* = 0.017), ADK (*P* < 0.0001) and patients with wild-type TP53 (*P* = 0.001). A negative correlation between NKX2–1 and miR-365 expression was found (*ρ* = −0.287; *P* = 0.003) but there was no correlation between NKX2–1 and miR-33a expression. Overall survival (OS) was longer in patients with high expression of NKX2–1 than in those with low expression (80.8 vs 61.2 months (*P* = 0.035), while a trend towards longer OS was observed in patients with low miR-365 levels (*P* = 0.07). The impact of NKX2–1 on OS and DFS was higher in patients with neither TP53 nor KRAS mutations. Higher expression of NKX2–1 was related to higher OS (77.6 vs 54 months; *P* = 0.017*)* and DFS (74.6 vs 57.7 months; *P* = 0.006) compared to low expression. The association between NKX2–1 and OS and DFS was strengthened when the analysis was limited to patients with stage I disease (*P* = 0.005 and *P=*0.003 respectively).

**Conclusions:**

NKX2–1 expression impacts prognosis in early-stage NSCLC patients, particularly in those with neither TP53 nor KRAS mutations.

**Electronic supplementary material:**

The online version of this article (10.1186/s12890-017-0542-z) contains supplementary material, which is available to authorized users.

## Background

Lung cancer is the leading cause of cancer-related death in developed countries [1, 2]. In Europe 410,000 new cases of lung cancer and 353,000 related deaths were estimated to occur in 2012 [3]. In the most frequent type, non-small-cell lung cancer (NSCLC), more than 70% of patients debut with locally advanced or metastatic disease [4]. In patients that present with early-stage disease, surgery alone or surgery followed by cisplatin-based adjuvant treatment is the first line of treatment. Although surgery is considered a curative treatment, 30–55% of patients will relapse during the first two years [5]. These data highlight the need to further investigate this disease and consolidate useful prognostic markers.

NK2 homeobox 1 (NKX2–1), also known as thyroid transcription factor-1 (TTF-1), is a key transcription factor that orchestrates the development of the lung, thyroid and forebrain in the embryonic period [6]. In adult lung tissue, NKX2–1 is expressed in conducting airways type II alveolar epithelial cells and in Clara cells and uniformly in the terminal respiratory unit [7]. NKX2–1 regulates surfactant protein transcription by directly binding to the promoter of SP-A [8], SP-B [9], SP-C [10], clara cell secretory protein (CCSP) [11], and T1〈 [12]. NKX2–1 is commonly used in clinical practice for the differential diagnosis of the adenocarcinoma (ADK) subtype of NSCLC [13, 14]. More than 70% of ADK are positive for NKX2–1 by immunohistochemistry, independent of disease stage [15].

Most of the studies that have examined the role of NKX2–1 in oncogenesis have highlighted its role as tumor suppressor. NKX2–1 inhibits proliferation by inhibiting the embryonal proto-oncogene High Mobility Group At-Hook 2 (HMGA2) [16]. In addition, NKX2–1 inhibits cell motility and metastatic capacity through modification of intercellular junctions and cytoskeleton elements. It promotes the expression of E-Cadherin, Ocludin (OCLN) and CLN 1/18 [17]. It also inhibits epithelial-to-mesenchymal transition (EMT) by repressing transforming growth factor β (TGF β), which produces an increase of E-Cadherin levels [18]. NKX2–1 also activates MYBPH synthesis, which inhibits actomyosin filaments, crucial elements in cell migration [19]. In addition, NKX2–1 regulates transcription of P53, a gene frequently lost or mutated in NSCLC patients [20, 21]. Reduced expression of NKX2–1 has been associated with initiation and progression of invasive mucinous lung ADK in patients harboring KRAS mutations [22].

MicroRNAs (miRNAs) are small non-coding RNAs (22–24 nucleotides in length) that regulate post-transcriptional processes via sequence-specific interactions with the 3′ untranslated regions (UTR) of mRNA [23]. Two miRNAs have been associated with NKX2–1: miR-365 and miR-33a. Using an in silico method, Mu et al. identified and validated miR-365 as a miRNA that directly regulates NKX2–1 by binding to its 3’UTR and inhibiting its translation [24]. Interestingly, a feed-back signaling loop between miR-365 and TGF β was described, illustrating that miR-365 participates in the NKX2–1 repression of TGF β [24]. miR-33a, located downstream of NKX2–1, was previously shown to have cholesterol homeostasis regulatory activity and to bind to the 3’UTR of HMGA2. These data indicate that NKX2–1 upregulates miR-33a, which represses HMGA2 [25] and could inhibit EMT, thus controlling lung cancer metastasis [26].

The prognostic value of NKX2–1 expression is controversial. Although most studies report that lower expression of NKX2–1 is associated with shorter overall survival (OS) in NSCLC [27–30], others report an inverse prognostic correlation [31]. We hypothesized that miR-365a, NKX2–1 and miR-33a could work together to influence growth and differentiation of lung cancer cells and that the study of this axis could thus help to understand the discrepancies observed in the prognostic impact of NKX2–1. We have studied the expression of NKX2–1 and its associated miRNAs, miR-365 and miR-33a, in a cohort of 110 early-stage NSCLC patients and correlated our findings with overall survival (OS).

## Methods

### Patient samples

From June 2007 to November 2013, tumor tissue samples were prospectively collected from 110 adult patients diagnosed with stage I-II NSCLC who underwent complete surgical resection in our institution. All patients had Spanish ethnicity. Tissue samples were immediately immersed in RNALater® (Ambion) and stored at −80 °C until processing. Clinical data were recorded on admission: age, gender, smoking history, preoperative pulmonary function tests, chronic obstructive pulmonary disease (COPD), Eastern Cooperative Oncology Group (ECOG) performance status (PS), clinical and postoperative staging according to TNM 7th edition [32], type of surgical resection and pathological findings (histological subtype, and the presence of emphysema). Information regarding adjuvant treatment, relapse and clinical outcomes was also recorded. The mutational status of TP53 and K-RAS was assessed in all patients, and EGFR mutational status was assessed in ADK patients.

### RNA extraction and gene expression analysis

Total RNA was isolated from frozen tissue using TriZol® Reagent (Life Technologies) according to the manufacturer’s protocol. RNA from samples was quantified using a NanoDrop ND-1000 Spectrophotometer (Fisher Scientific, Madrid, Spain).

cDNA was obtained from 500 ng of total RNA using the High Capacity cDNA Reverse Transcription Kit® (Life Technologies) as per manufacturer’s protocol. NKX2–1 mRNA expression levels were quantified using a TaqMan Gene Expression assay (Hs00968940_m1) in a 7500 Real-Time PCR System (Life Technologies). Relative expression levels were calculated by 2^-ΔΔCt^ method using 18S as endogenous control.

### miRNA quantification

miR-365 and miR-33a expression was analyzed using TaqMan MicroRNA Assay (Applied Biosystems) as previously described [33]. Relative quantification was calculated using 2^-ΔΔCt^. Normalization was performed with miR-191 [34]. All experiments were performed in triplicate.

### TP53, K-RAS and EGFR mutation analysis

PCR to identify TP53, K-RAS and EGFR mutations was performed on 50-ng DNA samples and the Sanger sequencing process was performed by STAB Vida (Caparica, Portugal). The mutation analysis for TP53 included the exons 5–8 and used the following primers: exon 5 forward(F) 5′- GTTTCTTTGCTGCCGTCTTC-3′, 5 reverse (R) 5’-GAGCAATCAGTGAGGAATCAGA-3′; exon 6F 5’-AGAGACGACAGGGCTGGTT-3′, 6R 5’-CTTAACCCCTCCTCCCAGAG-3′; exon 7F 5’-TTGCCACAGGTCTCCCCAA-3′, 7R 5’-AGGGGTCAGAGGCAAGCAGA-3′; exon 8F 5’-GGGACAGGTAGGACCTGATTT-3′, 8R 5’-TAACTGCACCCTTGGTCTCC-3′.

The mutation analysis for K-RAS included the codons 12 and 13 and used the following primers: F 5’-TTAACCTTATGTGTGACATGTTCTAA-3′, R 5’-AGAATGGTCCTGCACCAGTAA-3′.

The mutation analysis for EGFR included the exons 18, 19, 20 and 21 and used the following primers: exon 18 F 5’-GCATGGTGAGGGCTGAGGT-3′, 18R 5’-TGCAAGGACTCTGGGCTCC-3′; exon 19 F 5’-TGCATCGCTGGTAACATCCA-3′, 19R 5’-GAAAAGGTGGGCCTGAGGTT-3′; exon 20F 5’-TCCTTCTGGCCACCATGC-3′, 20R 5’-TGGCTCCTTATCTCCCCTCC-3′; exon 21F 5’-ATGCAGAGCTTCTTCCCATGA-3′, 21R 5’-CAGGAAAATGCTGGCTGACC-3′.

### TTF-1 immunohistochemistry staining

IHC was performed on formalin-fixed, paraffin-embedded tissue sections of 16 lung carcinomas and 3 normal lung controls from the Pathology Service of the Hospital Clinic of Barcelona after review by a thoracic pathologist. 4-μm-thick transverse sections of formalin-fixed, paraffin-embedded tissue were serially cut and mounted onto Dako Silanized Slides (S3003; Dako, Glostrup, Denmark). For antigen retrieval, the sections were manually immersed in Target Retrieval solution, high pH (Dako) and heated in a water bath at 95–99uC for 20 min. Endogenous peroxidase activity was quenched by immersion in Dako Real Peroxidase-Blocking solution for 10 min. The tissue sections were incubated with primary antibody against TTF1 (1:100, 8g7g3/1, DAKO, glostrop, Denmark). The slides were then washed in Tris–HCl and detected with horseradish peroxidase-conjugated anti-rabbit EnVision + kit (DAKO). Finally, sections were stained with hematoxylin. All slides were blindly scored by the same two pathologists. Nuclear staining of tumor cells was considered TTF1+. Tumors with completely no TTF1 expression in nuclei were de ned as TTF1 − .

### Statistical analyses

The primary endpoint of the study was OS, defined as the time between surgery and death from any cause. Kaplan-Meier curves for OS were drawn and compared by means of a log-rank test. All factors with *P* ≤ 0.1 in the univariate analysis were included in the Cox multivariate regression analyses for OS. Optimal cut-offs of NKX2–1 expression data for OS were assessed by means of maximally selected log-rank statistics using the Maxstat package (R statistical package, v. 2.8.1, Vienna, Austria) [35] and confirmed by the Kaplan-Meier test. Student T-Test or Mann-Whitney U test, as appropriate, were used for comparisons between two groups or ANOVA when more than two groups were compared. Pearson correlation was used to compare the NKX2–1 expression with its associated miRNAs. All statistical analyses were performed using PASW Statistics 21 (SPSS Inc.) and R v2.8.1. The level of significance was set at *P* ≤ 0.05.

## Results

### Patients

The analysis included 110 patients. The mean age was 66 years (range 32–84) and 79.1% were male. All patients had ECOG PS 0 or 1. The majority were current or former smokers. ADK was the most frequent histological subtype (51.8%) and SCC was the second most frequent (40%). Other subtypes included large-cell carcinoma (4.5%), mixed adenosquamous carcinoma (1.8%), and lepidic ADK (1.8%). The majority of patients had stage I disease (73.6%). Twenty-six patients (23.6%) received adjuvant chemotherapy, 19 for stage II and 7 for stage I disease with *T* > 4 cm. The mean follow-up was 45 months (range, 7.6–97.7). K-RAS mutations were detected in 19.1% of all patients and TP53 mutations in 30%. The distribution of TP53 mutations by exon was: 12 mutations in exon 5; six in exon 6; five in exon 7; and ten in exon 8. EGFR mutations were detected in 24.1% of patients with ADK (Table 1).

**Table 1:** Main patient characteristics

Characteristics	Value	*N* = 110	OS	NKX2–1
		*N* (%)	*p*-value	*p*-value
Sex	Male	87 (79.1)	0.019	0.196
	Female	23 (20.9)		
Age, yrs	Mean (Range)	66.1 (32–84)	0.018	0.181
	<=65	51 (46.4)		
	>65	59 (53.6)		
ECOG PS	0	20 (18.2)	0.067	0.109
	1	90 (81.8)		
Stage	I	81 (73.6)	0.334	0.429
	II	29 (26.4)		
Histology	ADK	57 (51.8)	0.325	*p* < 0.001
	SCC	44 (40)		
	Large-cell Carcinoma	5 (4.5)		
	Lepidic ADK	2 (1.8)		
	Mixed adenosquamous	2 (1.8)		
Type of surgery	Lobectomy/Bilobectomy	94 (85.5)	0.973	–
	Pneumonectomy	5 (8.1)		
	Atypical Resection	7 (6.4)		
Smoking history	Current Smoker	40 (36.3)	0.075	0.025
	Former Smoker	58 (52.7)		
	Never smoker	12 (10.9)		
Adjuvant treatment	Yes	26 (23.6)	0.663	–
	No	84 (76.4)		
Relapse	No	76 (69.1)	–	0.794
	Yes	34 (30.9)		
Emphysema	Yes	54 (49.1)	0.223	0.389
	No	49 (44.5)		
	Unknown	7 (6.4)		
COPD	Yes	71 (64.5)	0.574	0.127
	No	39 (35.5)		
DLCO (%)	Mean (Range)	73 (35–100)	–	–
Molecular Features	TP53 mutation	33/104 (30)	0.680	0.001
	KRAS mutation	21/109 (19.1)	0.815	0.204
	EGFR^a^	14/58 (24.1)	0.109	0.696

*OS* overall survival, *ECOG PS* Eastern Cooperative Oncology Group performance status, *ADK* adenocarcinoma, *SCC* squamous cell carcinoma, *COPD* chronic obstructive pulmonary disease, *DLCO* diffusing capacity of the lung for carbon monoxide

^a^EGFR mutational status was assessed only in adenocarcinoma patients

### NKX2–1, miR-365 and miR-33a expression

**Fig. 1:**
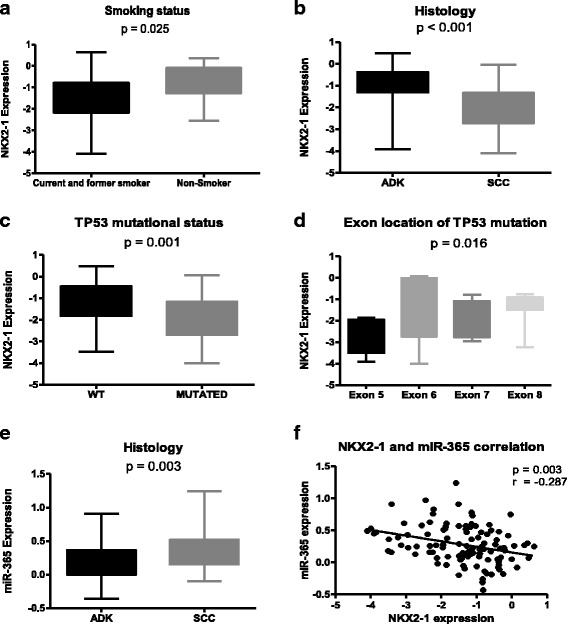
NKX2–1 and miR-365 expression. NKX2–1 expression according to **a** smoking status, **b** histology, **c** TP53 mutational status, and **d** exon location of TP53 mutation. **e** miR-365 expression according to histology. **f** Pearson correlation graph of NKX2–1 and miR-365 expression

NKX2–1 was differentially expressed according to the smoking status of the patient (ANOVA *P* = 0.03) and was upregulated in never-smokers compared to current and former smokers (*P* = 0.025; Fig. 1a).

NKX2–1 expression was also significantly higher in patients with ADK than in those with squamous cell carcinoma (SCC) (*P* < 0.001; Fig. 1b). A random selection of samples with ADK were assessed by IHC for NKX2–1. NKX2–1 expression was higher in ADK patients who were IHC-positive for NKX2–1(*P =* 0.043; Additional file 1: Figure S1).

Patients harboring TP53 mutations had lower NKX2–1 levels than those with wild-type (WT) TP53 (*P* = 0.001; Fig. 1c). ANOVA analysis showed that NKX2–1 was differentially expressed in TP53-mutated patients according to exon location (*P* = 0.02; Fig. 1d). No significant differences in NKX2–1 expression were observed according to KRAS or EGFR mutational status. miR-365 expression was significantly lower in ADK than in SCC patients (*P* = 0.003; Fig. 1e).

When we assessed the potential correlation between the expression of NKX2–1, miR-365, and miR-33a, expression, we found only a negative correlation between NKX2–1 and miR-365 expression (*ρ* = −0.287, *P* = 0.003; Fig. 1f).

### The prognostic value of NKX2–1, miR-365 and miR-33a in early-stage NSCLC

**Fig. 2:**
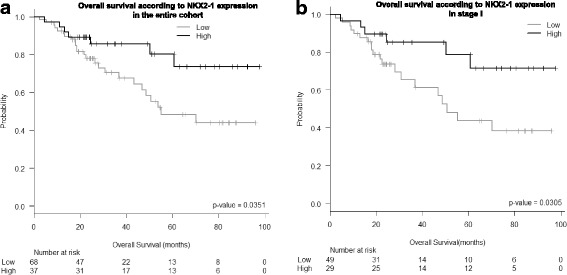
Kaplan Meier analysis of overall survival according to NKX2–1 expression levels in **a** the entire cohort and **b** patients with stage I disease

Using the optimal cutoff identified by Maxstat, patients were classified as having high or low NKX2–1 expression. Mean OS in patients with high NKX2–1 expression was 80.8 months (95% CI 69.6–92), compared to 61.2 months (95% confidence interval [CI] 51.3–71.1) in those with low NKX2–1 expression (*P* = 0.035; Fig. 2a). In addition, patients with high NKX2–1 expression had longer DFS than those with low expression levels (71.7 vs 52.1 months; *P* = 0.073; Additional file 1: Figure S2A) When the analysis was limited to patients with stage I disease, mean OS was 79.7 months (95% CI 67.1–92.3) in patients with high NKX2–1 expression and 57.0 months (95% CI 45.2–68.9) in those with low NKX2–1 expression (*P* = 0.031; Fig. 2b).

Patients with high levels of miR-365 showed a trend toward shorter OS than those with high levels (entire cohort: 60.9 vs 79.2 moths, *P* = 0.073; Stage I disease: 58.3 vs 76.6 months, *P* = 0.118; Additional file 1: Figure S3). No association between miR-33a and OS was observed.

### The prognostic value of NKX2–1 according to TP53, KRAS and EGFR mutational status

Since NKX2–1 has previously been linked to TP53 [20], KRAS [22] and EGFR [36], we explored its prognostic impact according to the mutational status of these genes in our entire cohort. No significant differences in OS according to NKX2–1 levels were found in patients with TP53, KRAS or EGFR mutations. However, in patients without TP53 mutations, OS for those with high NKX2–1 expression was 80.36 months (95% CI 68.1–92.6), compared to 56.4 months (95% CI 43.3–67.5) for those with low NKX2–1 expression (*P* = 0.048; Additional file 1: FigureS4A). In patients without KRAS mutations, high NKX2–1 expression was also associated with longer OS. OS for those with high NKX2–1 expression was 79.9 months (95% CI 68.7–91.1), compared to 60.2 months (95% CI 49.3–71.1) for those with low NKX2–1 expression (*P* = 0.027; Additional file 1: Figure S4B). These associations were maintained when the analysis was limited to stage I patients (TP53, *P =* 0.019; KRAS: *P =* 0.020; Additional file 1: FigureS5A–D). In patients without EGFR mutations, there was no association between NKX2–1 expression and OS.

**Fig. 3:**
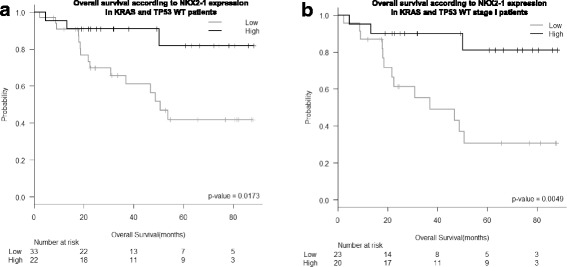
Kaplan Meier analysis of overall survival according to NKX2–1 expression levels in patients harboring neither TP53 nor KRAS mutations in **a** the entire cohort and **b** patients with stage I disease

Among patients with neither TP53 nor KRAS mutations, the prognostic impact of NKX2–1 expression was even greater. OS was 77.6 months (95% CI 66.4–88.7) for patients with high NKX2–1 expression and 54 months (95% CI 42.1–65.9) for those with low expression (*P* = 0.017; Fig. 3a). DFS was 74.6 months (95% CI 61.5–87.8) for patients with high NKX2–1 expression and 45.35 months (95% CI 22–57.7) for those with low expression (*P =* 0.006; Additional file 1: FigureS2B). This association was maintained when the analysis was limited to stage I patients in OS (76.9 [95% CI 65.1–88.7] vs 46.1 [95% CI 32.3–59.8] months, respectively; *P* = 0.005; Fig. 3b), and DFS (73.5 [95% CI 59.5–87.6] vs 39.6 [95% CI 26–53.2] months, respectively; *P* = 0.003).

In the multivariate analysis of all patients without TP53 or KRAS mutations, including age, sex, disease stage, adjuvant therapy, and NKX2–1 expression, NKX2–1 expression emerged as an independent prognostic factor for OS (OR 5.335, 95% CI 1.329–21.421; *P* = 0.018), together with stage (*P* = 0.018) and adjuvant therapy (*P* = 0.048).

In a multivariate analysis NKX2–1 emerges as the only independent prognostic factor for DFS (OR 4.333, 95% CI 1.466–12.807; *P =* 0.008).

## Discussion

The potential prognostic impact of NKX2–1 in NSCLC is unclear. Several studies have found an association between low NKX2–1 expression and good prognosis [27–30, 37, 38] while others have reported an association with poor prognosis [39, 40] and still others have found no association at all [41–43]. In the present study, we assessed NKX2–1 expression in a cohort of 110 patients with stage I-II NSCLC who had undergone surgical resection as their first therapeutic approach. Our findings indicate that high NKX2–1 expression is associated with longer OS both in the entire cohort (*P* = 0.035) and in the subgroup of stage I patients (*P* = 0.031). Moreover, among patients with neither TP53 nor KRAS mutations, NKX2–1 expression emerged as an independent prognostic factor for OS (OR 5.335; *P* = 0.018) and DFS (OR 4.333, *P =* 0.008).

The conflicting findings of previous studies may be due to several factors, including the techniques used for NKX2–1 analysis and the heterogeneity of patient cohorts.

Most of the studies evaluated NKX2–1 expression by Immunohistochemistry [38], a technique that only differentiates between the presence or absence of a protein but cannot quantify the levels of expression, although in some studies [30] the authors use an automated quantitative analysis of protein concentration within subcellular compartments to establish a range of expression. We have evaluated NKX2–1 levels using RT-PCR, which is a highly sensitive technique that can better classify patients with intermediate levels of a gene.

Arguably, the most important difference between studies of the prognostic impact of NKX2–1 lies in the clinical characteristics of the patients included, especially disease stage. A meta-analysis of 2235 patients included in 17 studies of NKX2–1 found that 937 patients (80%) had stage IIIb-IV disease [38]. In fact, the few studies reporting a negative prognostic impact for high NKX2–1 expression were performed in cohorts enriched in stage IIIb patients. Since the inclusion of these patients with locally advanced disease may well confound the identification of prognostic markers, we focused our study on a well-characterized cohort of early-stage patients. Our results are in line with other previous studies [27] where high NKX2–1 expression was related to longer survival. Interestingly, this association was maintained in the subgroup of stage I NSCLC patients, who had not received adjuvant treatment after surgery, suggesting a clear prognostic role for NKX2–1 mRNA expression.

NKX2–1 expression was upregulated in our patients with wild-type TP53. This can be explained by previous findings that NKX2–1 has been linked to regulation of TP53 transcription via LKB1 loss and IKKβ/NF-κB activation [20, 44].

. *NKX2–1* gene amplification displayed a positive correlation with the presence of KRAS mutations; however, its prognostic impact remains controversial. In a Japanese ADK cohort, *NKX2–1* amplification was an independent predictor of poor prognosis [45, 46] NKX2–1 has also been linked to KRAS in that NKX2–1 gene haploinsufficiency in patients harboring KRAS mutations, with a consequent loss of function of NKX2–1, may promote tumorigenesis in mucinous ADK [47]. In our patients, however, we found no significant differences in NKX2–1 expression according to KRAS status. Nevertheless, when we analyzed the effect of NKX2–1 expression on OS in patient subgroups classified according to TP53 and KRAS mutation status, we found a remarkable impact among patients with both wild-type TP53 and wild-type KRAS. OS in these patients was much longer for patients with high NKX2–1 levels – both in the entire cohort (*P* = 0.017) and in stage I patients (*P* = 0.005). Furthermore, in an exploratory analysis in this subgroup of patients, we found a strong association between high NKX2–1 expression and longer disease-free survival – again both in the entire cohort (*P* = 0.006) and in patients with stage I disease (*P* = 0.003). These findings suggest that NKX2–1 functions are linked to the normal functioning of TP53 and KRAS and that mutations in these genes influence the prognostic impact of NKX2–1 expression.

As expected, NKX2–1 expression in our cohort was higher in ADK than in SCC patients, although high NKX2–1 expression has previously been described in SCC as well [48]. Moreover, we observed an inverse correlation between smoking habit and NKX2–1 levels that is in line with previous reports [49, 50], suggesting that NKX2–1 downregulation could be related to smoking habits.

The underlying mechanism of NKX2–1 as a tumor suppressor is not fully understood but we speculated that it might be linked to its associated miRNAs, miR-365 and miR-33a. We found a negative correlation between miR-365 and NKX2–1 expression, indicating that miR-365 directly regulates NKX2–1. We also observed an inverse relation between miR-365 and NKX2–1 expression in ADK vs SCC, where miR-365 expression was lower in ADK patients while NKX2–1 expression was higher. Moreover, patients with high levels of miR-365 showed a trend towards shorter OS (*P* = 0.073). Taken together, these findings provide further evidence for a regulatory role of miR-365 over NKX2–1. In contrast, no significant correlation between NKX2–1 and miR-33a was observed, suggesting that factors other than NKX2–1 are involved in the regulation of miR-33a.

## Conclusions

The present study confirms a prognostic role for NKX2–1 in early-stage resected NSCLC patients, particularly in those harboring neither TP53 nor KRAS mutations. NKX2–1 expression may thus prove useful for the risk stratification of early-stage NSCLC patients and could be considered when deciding on adjuvant therapy strategies.

## Additional file

Additional file 1: Figure S1. NKX2–1 expression according to TTF-1 immunohistochemistry. **Figure S2.** Kaplan Meier analysis of disease-free survival according to NKX2–1 expression levels in **(A)** the entire cohort and **(B)** patients with stage I disease. **Figure S3.** Kaplan Meier analysis of overall survival according to miR-365 expression levels in **(A)** the entire cohort and **(B)** patients harboring neither TP53 nor KRAS mutations. **Figure S4.** Kaplan Meier analysis of overall survival according to NKX2–1 expression levels in **(A)** patients with wild-type TP53, **(B)** patients with TP53 mutations, **(C)** patients with wild-type KRAS, and **(D)** patients with KRAS mutations. **Figure S5.** Kaplan Meier analysis of the impact of NKX2–1 in overall survival in stage I disease **(A)** WT TP53 patients, **(B)** TP53 mutated patients, **(C)** KRAS WT patients, and **(D)** KRAS mutated patients. (PDF 276 kb)

## Abbreviations

ADK: Adenocarcinoma
CI: Confidence interval
COPD: Chronic obstructive pulmonary disease
DFS: Disease free survival
ECOG: Eastern Cooperative Oncology Group
EMT: Epithelial mesenchymal transition
miRNA: microRNA
NSCLC: Non-small-cell lung cancer
OS: Overall survival
PS: Performance status
SCC: Squamous cell carcinoma
UTR: Untranslated region
