# Supplementary material for: NKX2–1 expression as a prognostic marker in early-stage non-small-cell lung cancer
Source: BMC Pulm Med. 2017 Dec 13;17:197. doi: 10.1186/s12890-017-0542-z (PMC5727907; doi:10.1186/s12890-017-0542-z)

Supplementary Figure 1

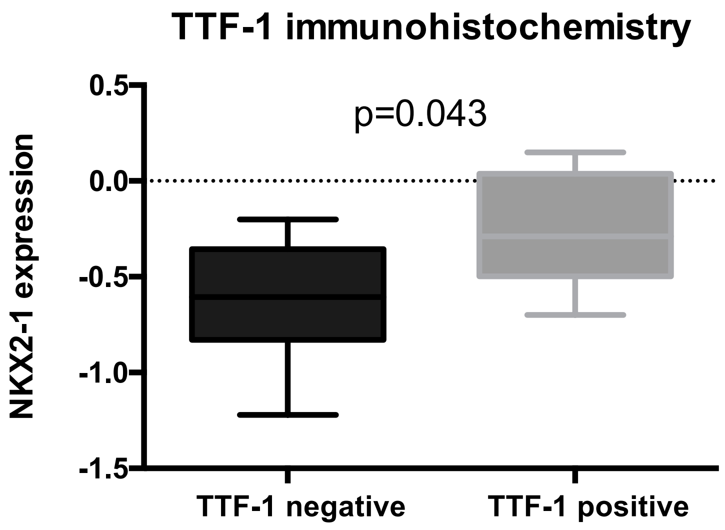

Supplementary Figure 2

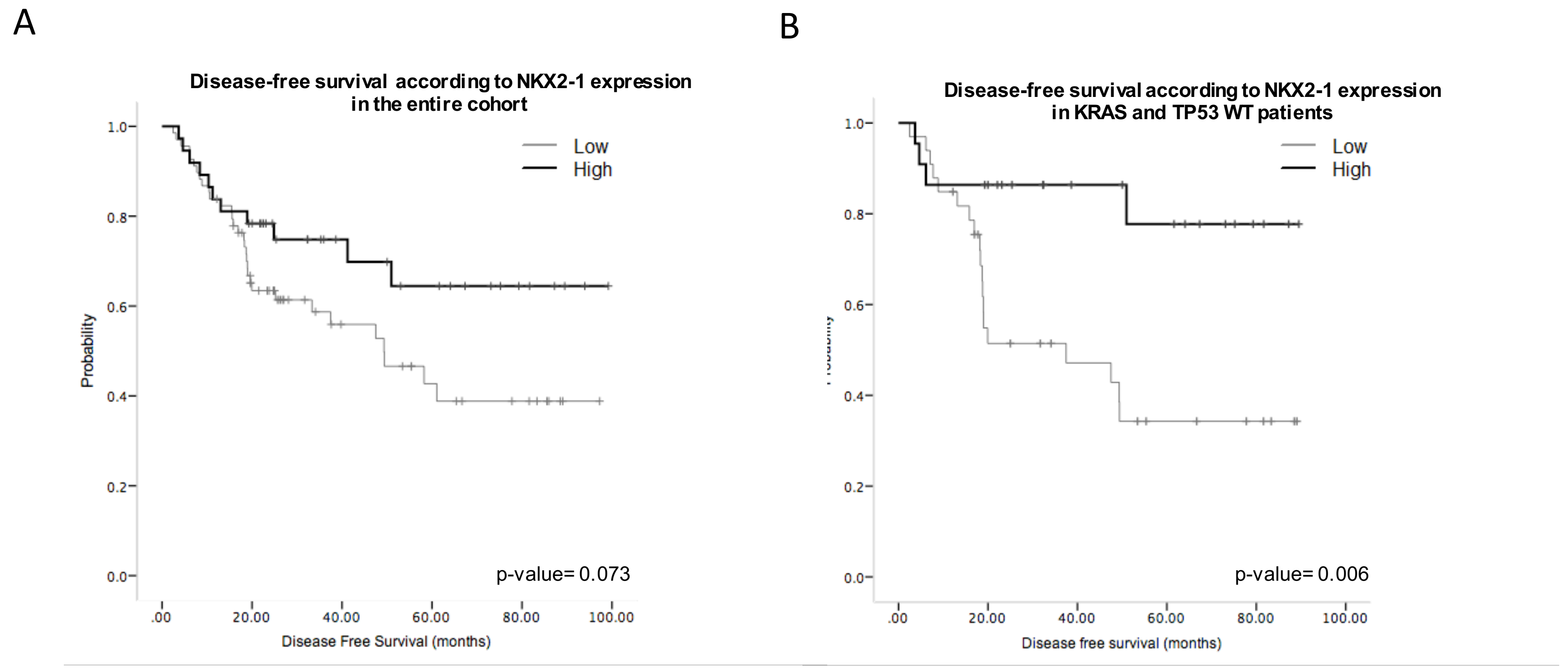

Supplementary Figure 3

A

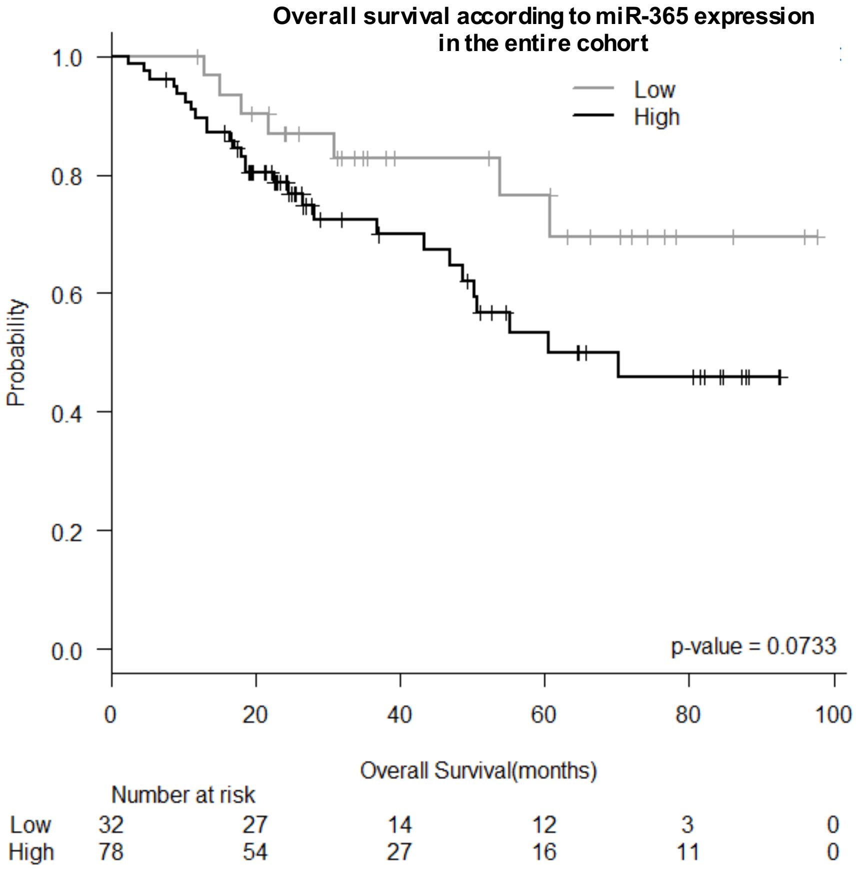

B

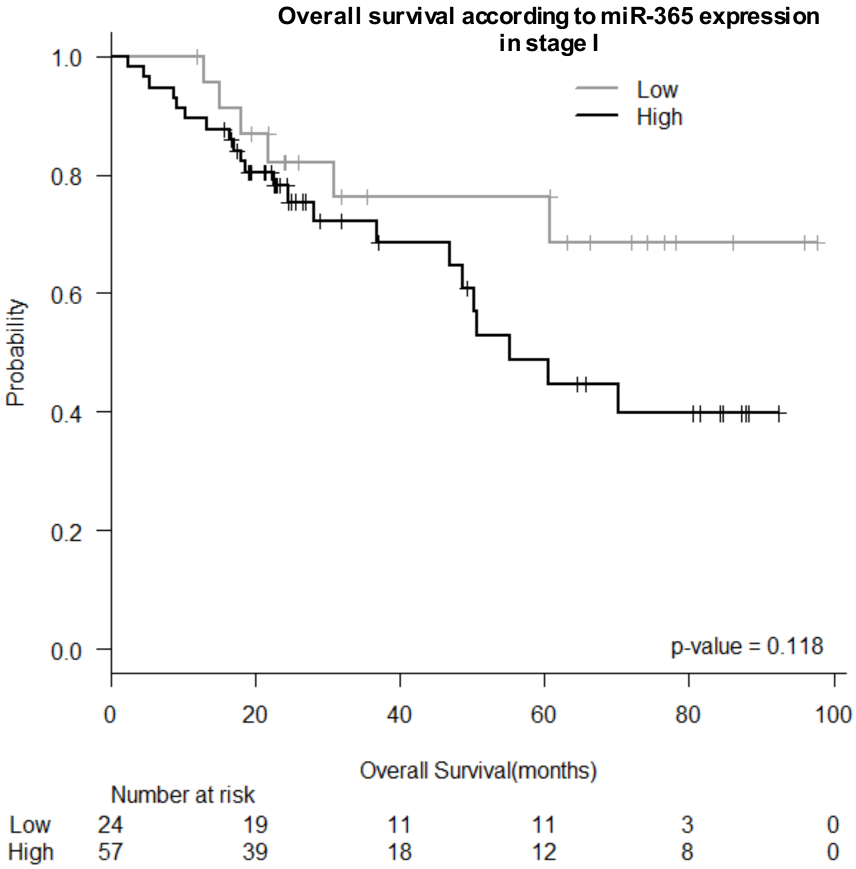

Supplementary Figure 4

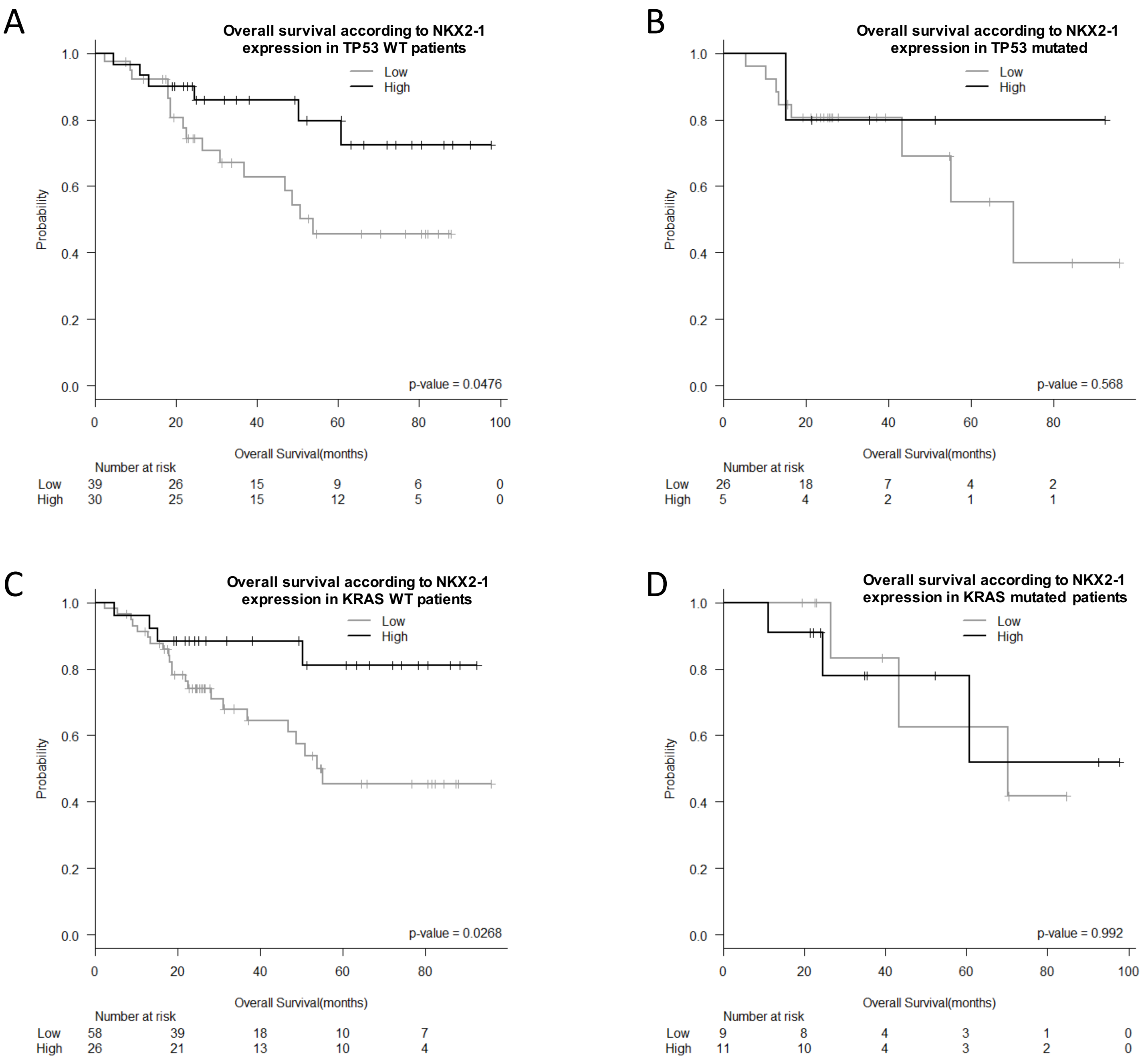

Supplementary Figure 5

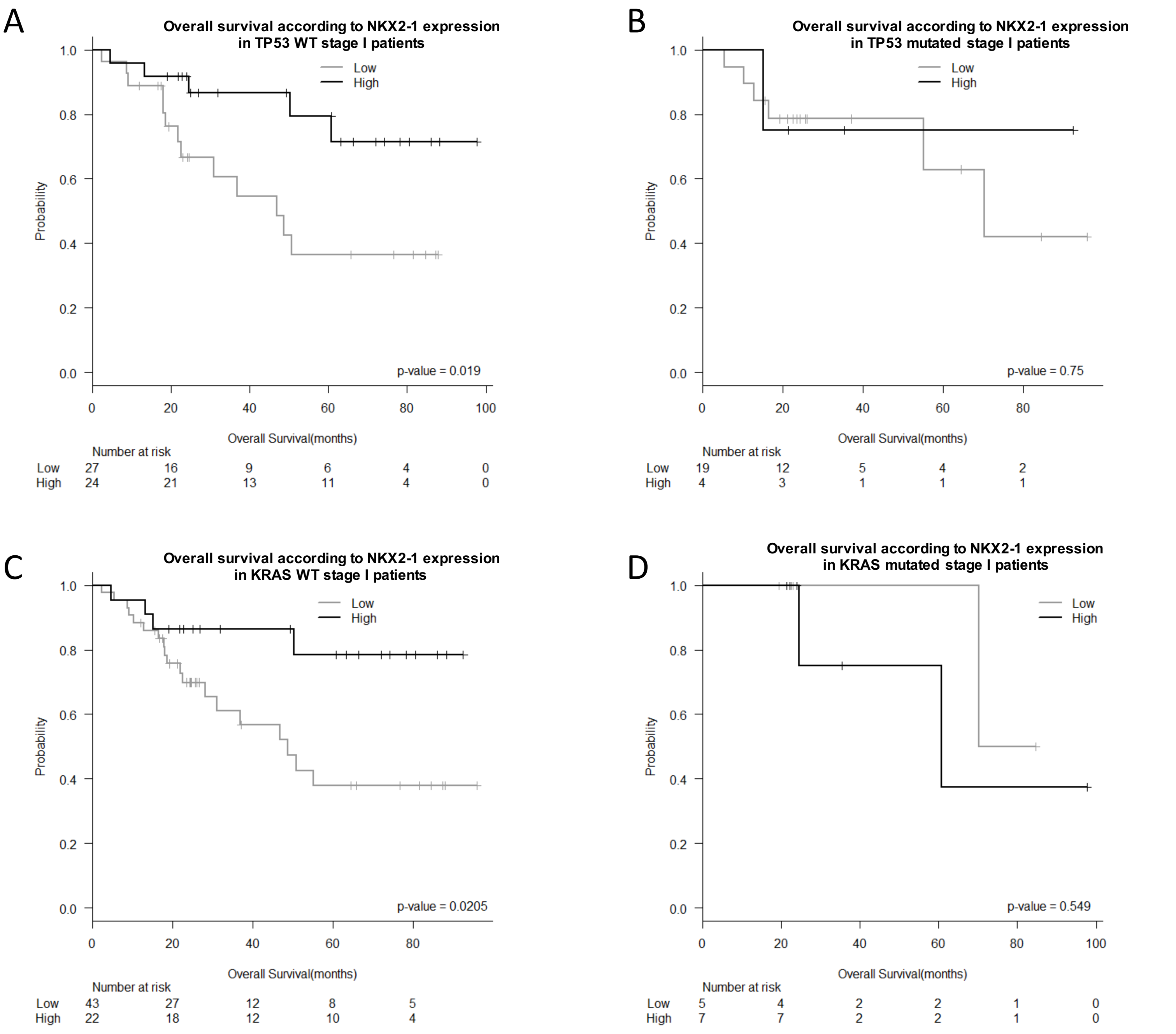

Supplement: Additional file 1: Figure S1. — NKX2–1 expression according to TTF-1 immunohistochemistry. Figure S2. Kaplan Meier analysis of disease-free survival according to NKX2–1 expression levels in (A) the entire cohort and (B) patients with stage I disease. Figure S3. Kaplan Meier analysis of overall survival according to miR-365 expression levels in (A) the entire cohort and (B) patients harboring neither TP53 nor KRAS mutations. Figure S4. Kaplan Meier analysis of overall survival according to NKX2–1 expression levels in (A) patients with wild-type TP53, (B) patients with TP53 mutations, (C) patients with wild-type KRAS, and (D) patients with KRAS mutations. Figure S5. Kaplan Meier analysis of the impact of NKX2–1 in overall survival in stage I disease (A) WT TP53 patients, (B) TP53 mutated patients, (C) KRAS WT patients, and (D) KRAS mutated patients. (PDF 276 kb) [file 12890_2017_542_MOESM1_ESM.pdf]
